# Abundance of *Bemisia tabaci* Gennadius (Hemiptera: Aleyrodidae) and its parasitoids on vegetables and cassava plants in Burkina Faso (West Africa)

**DOI:** 10.1002/ece3.4078

**Published:** 2018-05-20

**Authors:** Rahim Romba, Olivier Gnankine, Samuel Fogné Drabo, Fidèle Tiendrebeogo, Hélène Henri, Laurence Mouton, Fabrice Vavre

**Affiliations:** ^1^ Laboratoire d'Entomologie Fondamentale et Appliquée, Unité de Formation et de Recherche en Sciences de la Vie et de la Terre (UFR‐SVT) Université Ouaga I Pr Joseph Ki Zerbo Ouagadougou Burkina Faso; ^2^ Laboratoire Mixte International Patho‐Bios IRD‐INERA Ouagadougou Burkina Faso; ^3^ Université de Lyon Université Lyon 1 CNRS Laboratoire de Biométrie et Biologie Evolutive UMR5558 Villeurbanne France

**Keywords:** abundance, *Bemisia tabaci*, host plants, natural enemies, parasitism

## Abstract

The whitefly *Bemisia tabaci* is a pest of many agricultural and ornamental crops worldwide and particularly in Africa. It is a complex of cryptic species, which is extremely polyphagous with hundreds of host plants identified around the world. Previous surveys in western Africa indicated the presence of two biotypes of the invasive MED species (MED‐Q1 and MED‐Q3) living in sympatry with the African species SSA and ASL. This situation constitutes one of the rare cases of local coexistence of various genetic entities within the *B. tabaci* complex. In order to study the dynamics of the distribution and abundance of genetic entities within this community and to identify potential factors that could contribute to coexistence, we sampled *B. tabaci* populations in Burkina Faso in 2015 and 2016 on various plants, and also their parasitoids. All four genetic entities were still recorded, indicating no exclusion of local species by the MED species. While *B. tabaci* individuals were found on 55 plant species belonging to eighteen (18) families showing the high polyphagy of this pest, some species/biotypes exhibited higher specificity. Two parasitoid species (*Eretmocerus mundus and Encarsia vandrieschei*) were also recorded with *E*. *mundus* being predominant in most localities and on most plants. Our data indicated that whitefly abundance, diversity, and rate of parasitism varied according to areas, plants, and years, but that parasitism rate was globally highly correlated with whitefly abundance suggesting density dependence. Our results also suggest dynamic variation in the local diversity of *B. tabaci* species/biotypes from 1 year to the other, specifically with MED‐Q1 and ASL species. This work provides relevant information on the nature of plant–*B. tabaci*‐parasitoid interactions in West Africa and identifies that coexistence might be stabilized by niche differentiation for some genetic entities. However, MED‐Q1 and ASL show extensive niche overlap, which could ultimately lead to competitive exclusion.

## 1 INTRODUCTION

Vegetable plants are cultivated in many places for their socioeconomic and nutritional importance. In Africa, the main vegetable plants belong to families of Asteraceae, Compositae, Cruciferae, Cucurbitaceae, Euphorbiaceae, Fabaceae, Leguminosae, Malvaceae, and Solanaceae. In sub‐Saharan Africa, cassava (*Manihot esculenta* Crantz, Euphorbiaceae) is also a major food staple crop (AVRDC, 2004) due to its high caloric content. Unfortunately, the production of cassava and vegetables is currently constrained by viral diseases and pest attacks, especially due to the whitefly *Bemisia tabaci* (Gennadius). This insect affects agricultural crops as well as ornamentals. More than 600 species of plants can be attacked by this pest in both field and glasshouse settings (Gelman, Gerling, Blackburn, & Hu, 2005). It causes damage directly by feeding on sap and, to an even greater extent, indirectly by transmitting several hundreds of plant viruses (Begomovirus, Crinivirus, Ipomovirus, Torradovirus), leading to high yield losses (Brown & Czosnek, 2002; Jones, 2003; Lapidot & Polston, 2010).

In West Africa, *B. tabaci* was first recorded on cassava in Côte d'Ivoire in 1931 (Hédin, 1931) and in Nigeria in 1936 (Golding, 1936). In 1998, outbreaks were recorded in cotton fields in Burkina Faso, Mali, and Côte d'Ivoire inducing severe crop damages (Otoidobiga, Vincent, & Stewart, 2003). The resulting losses severely impacted the economic activity of these countries (Gnankiné, Traore, Sanon, Traore, & Ouédraogo, 2007). Nowadays *B. tabaci* has been reported in all tropical and subtropical regions.

In order to understand outbreaks and better control *B. tabaci* populations, a key element to keep in mind is the biodiversity encountered in this species complex. Indeed, phylogenetic studies based on the mitochondrial gene *COI* revealed that *B. tabaci* is a complex of at least 41 cryptic species that belong to 11 major genetic groups (Ahmed, De Barro, Ren, Greeff, & Qiu, 2013; Bing, Ruan, Rao, Wang, & Liu, 2013; De Barro, Liu, Boykin, & Dinsdale, 2011; Dinsdale, Cook, Riginos, Buckley, & De Barro, 2010; Hu et al., 2011; Liu, Colvin, & De Barro, 2012; Sun, Xu, Luan, & Liu, 2011; Wang, Sun, Qiu, & Liu, 2011). Among these putative species, the Middle East–Asia Minor 1 (MEAM1), commonly known as biotypes B and B2, and the Mediterranean (MED), which regroups the biotypes MED‐Q, MED‐J, and MED‐L, are the most invasive ones and predominate in many areas (Dinsdale et al., 2010). According to De Barro et al. (2011), ASL also belongs to the MED species. However, population genetic analyses support that ASL (Africa Silver Leafing) is a distinct species (Mouton et al., 2015), and thus, it will be considered as such in this study. Within the MED‐Q biotype, genetic diversity has been observed leading to the recognition of three *mtCOI* haplotypes, MED‐Q1, MED‐Q2, and MED‐Q3 (Chu et al., 2012; Gueguen et al., 2010). Five putative species of *B. tabaci*, namely the sub‐Saharan African SSA1 to SSA5 (Legg, Shirima et al., 2014), have also attracted particular attention in the last 10 years because they have been recognized as important pest vectors of several key African cassava viruses inducing severe yield losses and food scarcity, such as the Cassava mosaic diseases (CMD) and the Cassava brown streak disease (CBSV; Legg et al., 2011). These species, within which biotypes can be distinguished, show diverse behaviors related to host plant preference, oviposition, ecological adaptation, and capacity of virus dissemination (De Barro, Trueman, & Frohlich, 2005; Perring, 2001). They also differ in the composition of the community of endosymbiotic bacteria they harbor (De Barro et al., 2011; Dinsdale et al., 2010; Gnankiné, Mouton, Henri et al., 2013; Gueguen et al., 2010).

Despite these different biological characteristics that should allow stable coexistence owing to niche differentiation, competitive exclusion of local species by MEAM1 or MED invaders has been repeatedly reported (Brown, Frohlich, & Rosell, 1995). Three main processes have been proposed to explain these exclusions: the broad host range of MEAM1 and MED species, their higher resistance to insecticides (that could also explain how MED has replaced MEAM1 in some part of the world), and reproductive interferences (Horowitz, Kontsedalov, Khasdan, & Ishaaya, 2005; Khasdan et al., 2005; Luan, De Barro, Ruan, & Liu, 2013; Moya, Guirao, Cifuentes, Beitia, & Cenis, 2001; Tsagkarakou, Tsigenopoulos, Gorman, Lagnel, & Bedford, 2007; Wang et al., 2011).

A remarkable situation has however been described recently in Burkina Faso, where the local ASL species was found in sympatry with two biotypes belonging to the MED species, MED‐Q1 and MED‐Q3, a biotype that has yet only been found in Burkina Faso (Gnankiné, Ketoh, & Martin, 2013; Mouton et al., 2015). Although data about their host range remain limited, MED‐Q1 and ASL seem much more generalists than MED‐Q3 that appears mostly restricted to *Lantana camara* (Gnankiné, Mouton, Henri et al., 2013), where neither MED‐Q1 nor ASL was found. In addition, the degree of resistance to insecticides also varies between them (Gnankiné, Mouton, Savadogo et al., 2013; Houndété et al., 2010). While still limited, these different elements could play a role in favoring or, on the opposite, in limiting coexistence.

In order to follow the dynamics of the *B. tabaci* community present in Burkina Faso and to identify potential factors involved in exclusion or coexistence patterns, we extended our sampling to two new years and encompassing a larger diversity of sites and plants to define more precisely the realized niche of the different genetic entities. We monitored not only presence, but also abundance, in order to assess potential competition among individuals. Because herbivores communities may also be affected by top‐down effects, we also monitored the presence of parasitoids and the rate of parasitism. Overall, these new data provide a sound basis to determine the stability of the *B. tabaci* community at the spatial and temporal scales.

## 2 MATERIALS AND METHODS

### 2.1 Sampled localities

**Figure 1:**
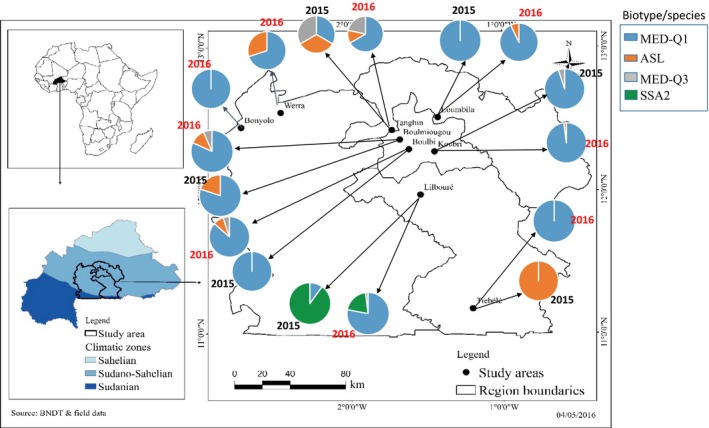
Map of the sampling areas in Burkina Faso and prevalence of *Bemisia tabaci* species/biotypes in 2015 and 2016

This study was conducted in Burkina Faso, located in the west part of the African continent (Figure 1). The climate is tropical with two seasons: the dry (from October to April) and the rainy (from May to September) seasons. This study was conducted during the dry season, the main season for vegetable production, from March to April, and two consecutive years (2015 and 2016) in nine localities in the provinces of Kadiogo (Tanghin, Boulmiougou, Boulbi, Koubri), Oubritenga (Loumbila), Nahouri (Tiébélé), Bazèga (Lilbouré), Boulkiemdé (Werra), and Sanguié (Bonyolo) that all belong to the Sudano‐Sahelian zone.

### 2.2 Sampling, determination of host plant species, *B. tabaci* abundance, and parasitism rate

#### 2.2.1 Sampling

The host plants of *B. tabaci* were surveyed in nine localities of Burkina Faso in 2015 and 2016. Plants included vegetables and ornamental plants as well as weeds. Only host plants where whitefly adults, eggs, and nymphs were found were considered. For these plants, five leaves (from top to bottom) were taken and examined in the laboratory under a binocular microscope. Identification of the insect larvae was carried out using the taxonomic keys of Martin (1987) and Martin, Mifsud, and Rapisarda (2000). The plant species were identified according to the key of Thiombiano (2012) and Akoégninou, Vander Burg, Vander, and Maessen (2006).

#### 2.2.2 Whitefly abundance

The estimation of *B. tabaci* abundance was performed on 12 plant species: vegetable crops, cassava, and *Lantana camara*. For cultivated plants, the surface of the parcels was about one‐quarter hectare for *Capsicum annuum, Capsicum frutescens, Cucurbita pepo, Cucumis sativus, Hibiscus esculentus, Solanum lycopersicum, Solanum aethiopicum, Solanum incanum,* and *Vigna unguiculata* and 1 ha for *Manihot esculenta* and *Ipomoea batatas*. For each plant species and parcel, thirty plants were randomly taken along two diagonal transects across each field (15 plants per diagonal) according to the method described in Sseruwagi, Sserubombwe, Legg, Ndunguru, and Thresh (2004). For *Lantana Camara*, the design sampling was different and thirty plants were selected randomly on a surface of about the quarter of hectare. As adults feed and oviposit preferentially on immature leaves, whitefly abundance was determined by the average number of adults on the five youngest apical leaves of each plant. Each leaf was held by the petiole and gently inverted in order to count the number of adults present on the lower surface (Avidov & Harpaz, 1969; Fargette, 1985). These adults were collected and preserved in 1.5‐ml tubes containing 70% ethanol.

#### 2.2.3 Parasitism rate

The rate of parasitism was evaluated on the same plants by dividing the number of *B. tabaci* nymphs that were parasitized by the total number of nymphs present on the five leaves. Parasitized nymphs can be identified by eyes through their color, black or dark yellow. The leaves with parasitized whitefly nymphs or pupae were collected, put into Petri dishes, and brought back to the laboratory. On the same plants, adult parasitoids of *B. tabaci* were also captured and placed directly into 1.5‐ml tubes containing 70% ethanol. Species of parasitoids were determined by Dr. Delatte (CIRAD, UMR PVBMT CIRAD‐Université de la Réunion).

### 2.3 Determination of whitefly species/biotypes by PCR‐RFLP

The species/biotype of 630 adult whiteflies was determined using a PCR‐RFLP method as described in Henri, Terraz, Gnankiné, Fleury, and Mouton (2013). This method proved to allow the identification of all the species/biotypes of *B. tabaci* present in western Africa. Briefly, DNA was extracted from each individual using the NucleoSpin Tissue kit (MACHEREY‐NAGEL). Elution was performed in 100 μl of BE buffer. Extracts were then stored at −20°C until use. The primers C1‐J‐2195 and L2‐N‐3014 were used to amplify a fragment of the *mtCOI* gene of *B. tabaci* with an approximate size of 800 bp (Simon et al., 1994). PCR amplifications were performed in a final volume of 25 μl containing 200 mmol/L dNTPs, 200 nmol/L of each primer, 1 mmol/L of MgCl 2, 0.5 U of DreamTaq, and 2 μl of DNA template. The cycling profile consisted of an initial denaturing phase at 94°C for 2 min, followed by 34 cycles consisting of 94°C for 30 s (denaturing), 58°C for 30 s (annealing), and 72°C for 1 min (extension), followed by a final extension phase at 72°C for 5 min. PCR products were digested with XapI and BfmI (10 UI) at 37°C for 3 hr, separated by electrophoresis on a 1.5% agarose gel at 100 V for 1 hr, and visualized by ethidium bromide staining.

### 2.4 Statistical analyses

Statistical analyses were performed with the R software version 3.2.2 (http://www.r-project.org/). The effects of the locality, the host plant species, and the year on the abundance of whiteflies and on the rate of parasitism were tested globally by ANOVA and also using Tukey's pairwise mean comparison tests. The distribution of species/biotype on host plants and sites was tested using Fisher's exact tests. A linear regression was conducted to test the relationship between the rate of parasitism and the total number of nymphs, and between the whitefly abundance in 2015 and that in 2016.

## 3 RESULTS

### 3.1 Host plants of *B. tabaci*

Our field surveys showed that the diversity of host plants of *B. tabaci* in Burkina Faso is high. In total, *B. tabaci* was found on 55 plant species belonging to 18 families were recorded (Table 1). Ten of the 55 species were ornamental, seventeen species were vegetables, and nineteen, six, and two species were weeds, strictly economic crops as defined by Li et al. (2011), such as cassava, tobacco, potato, and fruits, respectively.

**Table 1:** Host plant species where *Bemisia tabaci* was found in Burkina Faso

No.	Family	Plant species	Host type^a^	Location^b^
1	Amaranthaceae	*Amaranthus hybridus* L.	*W*	G
2		*Amaranthus viridis* L.	W	G
3	Asteraceae	*Ageratum conyzoides* L.	W	B
4		*Lactuca sativa* L. [cult.]	V	G, F, E, D
5	Brassicaceae	*Brassica oleracea* L. [cult.]	V	B, F, E, D
6	Capparaceae	*Cleome gynandra* L.	*O*	G, C
7		*Crateva adansonii* DC.	*F*	H
8	Caricaceae	*Carica papaya* L. [cult.]	F	G
9	Convolvulaceae	*Ipomoea batatas* (L.) Lam. [cult.]	E	G
10		*Ipomoea eriocarpa* R. Br.	*W*	C
11		*Ipomoea involucrata* P. Beauv.	*W*	G
12	Cucurbitaceae	*Cucumis sativus* L. [cult.]	V	C, B, C, D, F
13		*Cucurbita pepo* L. [cult.]	V	B, C
14		*Cucumis melo* L. [cult.]	V	H
15	Euphorbiaceae	*Euphorbia heterophylla* L.	O	G, H
16		*Jatropha gossypiifolia* L. [cult]	O	C
17		*Manihot esculenta* Crantz [cult.]	E	G, H
18		*Euphorbia hirta* L.	W	H
19	Fabaceae	*Gliricidia sepium* (Jacq.) Walp.	*O*	G
20		*Albizia lebbeck* (L.) Benth. [cult.]	*E*	G
21		*Cassia alata* L.	*O*	C
22		*Cassia occidentalis* L.	*E*	H
23		*Cassia obtusifolia* L.	*W*	G, B, C, F
24		*Phaseolus vulgaris* L. [cult.]	V	B, F
25		*Piliostigma reticulatum* (DC.) Hochst.	W	G
26		*Vigna unguiculata* (L.) Walp. [cult.]	V	H
27		*Vigna racemosa* (G.Don) Hutch.& Dalziel	W	H
28	Malvaceae	*Abelmoschus esculentus* (L.) Moench [cult.]	*V*	G, B, F, C
29		*Corchorus tridens* L.	V	G, B, F, C
30		*Hibiscus cannabinus* L. [cult.]	*W*	B, F, H
31		*Hibiscus sabdariffa* L. [cult.]	V	A, B, C, D, G,
32		*Corchorus olitorius* L.	W	H
33		*Waltheria indica* L.	*W*	H
34		*Triumfetta rhomboidea* Jacq.	*W*	H
35		*Hibiscus esculentus*	V	B, C, D, E, G
36		*Hibiscus scotellii* Baker f.	*W*	H
37		*Hibiscus diversifolius* Jacq.	*W*	H
38		*Hibiscus lobatus* (Murray) Kuntze	*W*	H
39		*Malvastrum coromandelianum* (L.) Garcke	*W*	G
40	Passifloraceae	*Passiflora edulis* Sims [cult.]	O	G
41	Phyllanthaceae	*Flueggea virosa* (Roxb. ex Willd) Voigt	*O*	G
42	Polygonaceae	*Polygonum salicifolium* Brouss. ex Willd	*W*	H
43	Portulacaceae	*Portulaca oleracea* L.	*V*	C
44	Rhamnaceae	*Ziziphus mauritiana* Lam.	*E*	G
45	Solanaceae	*Capsicum annum* L. [cult.]	V	G, B, F, C
46		*Capsicum frutescens* L. [cult.]	V	G, F, C
47		*Datura innoxia* Mill.	O	G.
48		*Solanum lycopersicum* L.	V	A, B, G,
49		*Nicotiana tabacum* L. [cult.]	E	C
50		*Physalis angulata* L.	W	G, B, C
51		*Solanum aethiopicum* L. [cult.]	V	G, B, D, E, F, C
52		*Solanum incanum* L.	V	G, B, D, E
53		*Solanum tuberosum* L. [cult.]	O	C, D, E
54	Verbenaceae	*Lantana camara* L.	O	G
55	Zygophyllaceae	*Balanites aegyptiaca* (L.) Delile	F	G, D

a E = economic crops; F = fruit; O = ornamental plants; V = vegetables; W = weeds.

b A, B, C, D, E, F, G, H, and I represent the localities of Bonyolo, Boulbi, Boulmiougou, Koubri, Lilbouré, Loumbila, Tanghin, Tiébélé, and Werra, respectively.

### 3.2 Whitefly abundance

**Figure 2:**
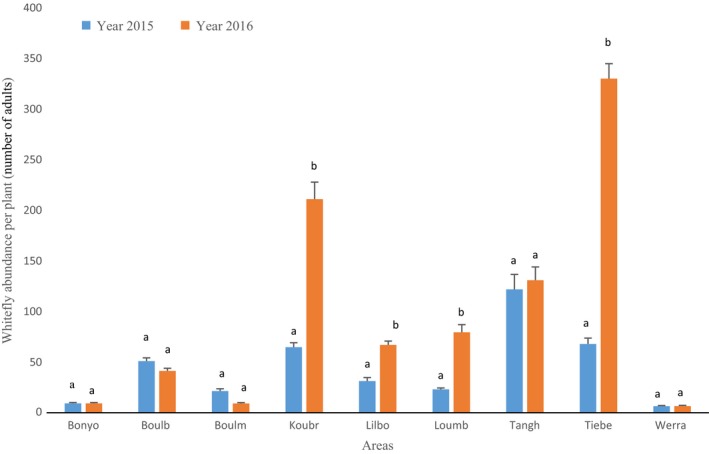
Average abundance of *Bemisia tabaci* in the different localities. Means followed by the same letters are not significantly different (generalized Tukey's all‐pair comparisons test at *p* < .05). Areas: Boulm: Boulmiougou; Bonyo: Bonyolo; Boulb: Boulbi; Koubr: Koubri; Lilbo: Lilbouré; Loumb: Loumbila; Tangh: Tanghin; Tiebe: Tiebélé

The mean whitefly abundance varied according to both the locality and the year, with a high interaction between these two factors (*F* = 66.97, *df* = 8, *p* value <2.10^−16^; Figure 2). The most infested areas were Tanghin, Tiébélé, Koubri, and Boulbi in 2015 and Tanghin, Tiébélé, Koubri, Loumbila, and Lilbouré in 2016.

**Figure 3:**
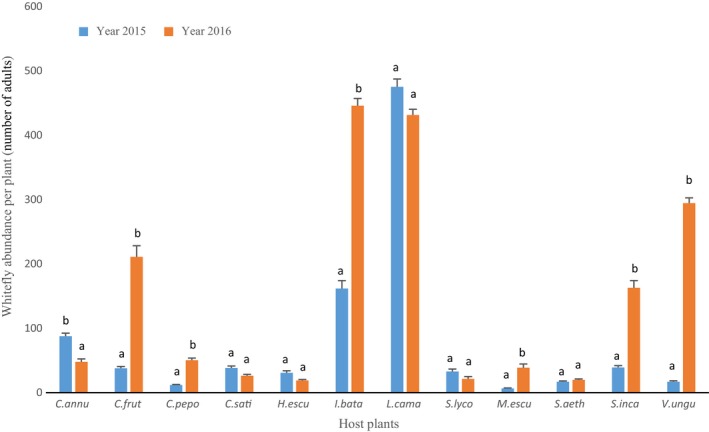
Average abundance of *Bemisia tabaci* on the host plants. Means followed by the same letters are not significantly different (generalized Tukey's all‐pair comparisons test at *p* < .05). Host plants: *C. annu: Capsicum annuum; C. frut: Capsicum frutescens, C. pepo: Cucurbita pepo; C. sati: Cucumis sativus; H. escu: Hibiscus esculentus; I. bata: Ipomoea batatas; L. cama: Lantana camara; S. lyco: Solanum lycopersicum; M. escu: Manihot esculenta; S. aeth: Solanum aethiopicum; S. inca: Solanum incanum; V. ungu: Vigna unguiculata*

There is also an interaction between the host plant and the year (*F* = 61.59, *df* = 11, *p* value <2.10^−16^; Figure 3). Host plants from the families Verbenaceae, Cucurbitaceae, Solanaceae, and Fabaceae were the most preferred hosts for *B. tabaci*. The whitefly abundance was the highest on *L. camara*,* I. batatas*, and *C. annum* in 2015 and *I. batatas*,* L. camara*,* C. frutescens*,* S. incanum*, and *V. unguiculata* in 2016.

Overall, the mean abundance increased significantly between 2015 and 2016, especially on *I. batatas, C. frutescens, S. incanum, and V. unguiculata*. In order to test whether the whitefly abundance exhibits some stability between years, a regression was performed according to the localities or the host plants. Results did not show any significant relationship per area between 2015 and 2016 (*r* ^2^ = .35, *p* = .08; Figure S1A). On the other hand, a significant linear correlation was detected for host plants (*r* ^2^ = .42, *p* = .01). However, this correlation was driven by two host plants, *L. camara* and *I. batatas*, that showed a very high abundance for the 2 years. When these two points were removed, the relationships became nonsignificant (*r* ^2^ = .005, *p* = .85; Figure S1B).

In conclusion, predicting abundance of *B. tabaci* is very difficult as it is highly variable across years, sites, and host plants.

### 3.3 Distribution of *B. tabaci* species/biotypes

#### 3.3.1 Geographic distribution

**Figure 4:**
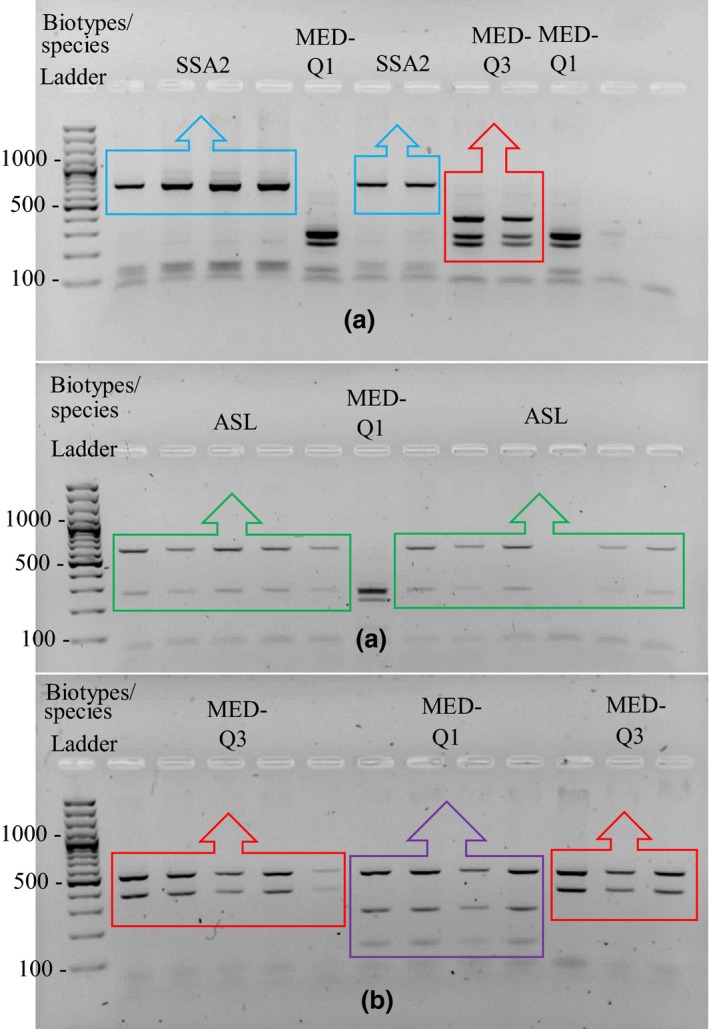
Profiles obtained on gel after PCR‐RFLP assays with XapI (a) and BfmI (b) to discriminate mitochondrial haplotypes. The size of the different bands obtained and the size (bp) of the bands corresponding to the ladder are indicated on the left of the figure

Among the 630 individuals sampled, three species were identified: ASL, the basal group SSA (sub‐Saharan African), and MED (Figure 4). Within the MED species, two biotypes were found, MED‐Q1 and MED‐Q3. Our results suggest that species and biotypes are not randomly distributed between sites (Fisher's exact test, *p* = .0005). The MED‐Q1 biotype was found in all the localities (except one, Tiébélé in 2015) where it predominated (Figure 1). ASL was found in several areas, but at lower frequencies (Table S1). MED‐Q3 and SSA2 were only found in a restricted number of localities, which could be explained by their host specialization. The frequency and the composition of the species/biotypes varied significantly from 2015 to 2016, but MED‐Q1 remained the most represented these two years (Fisher's exact test, *p* = 2.08.10^−11^). In some places, as in Lilbouré and Tiébélé, the proportion of MED‐Q1 increased greatly between 2015 and 2016 from 10% to 90% and from 0% to 100%, respectively (Table S1).

#### 3.3.2 Distribution on host plants

**Figure 5:**
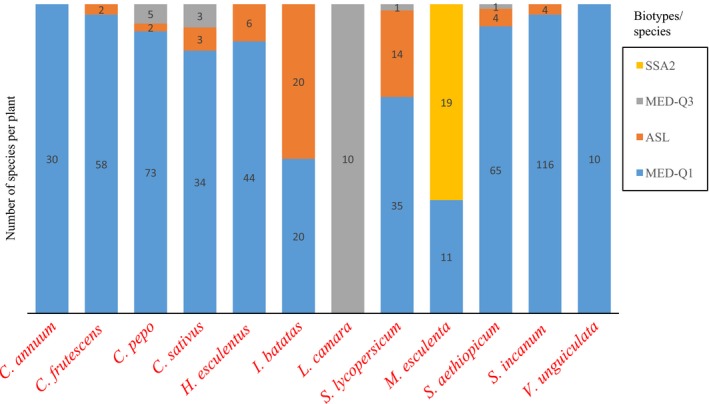
Distribution of *Bemisia tabaci* species/biotypes on plants (590 individuals in total). The numbers indicate the sample size

A significant relationship was found between the plant and the species/biotypes distribution (Fisher's exact test, *p* = .0005). Globally, most of the vegetables were infested by MED‐Q1 which was sometimes associated with ASL, MED‐Q3, and SSA2 (Figure 5). MED‐Q3 was the only biotype detected on *Lantana camara*. Interestingly, this is the plant that showed the highest abundance of *B. tabaci* in 2015 as well as in 2016. MED‐Q3 was also found in sympatry with MED‐Q1 on *Cucurbita pepo* and *Cucumis sativus* in Boulmiougou and on *Solanum aethiopicum* in Koubri, even though at very low frequency. SSA2 was the only group restricted to a single plant, cassava, where it was found together with MED‐Q1. These results highlight the important variation in host plant exploitation between *B. tabaci* species, but also within species.

### 3.4 Larval parasitism of *B. tabaci*

**Figure 6:**
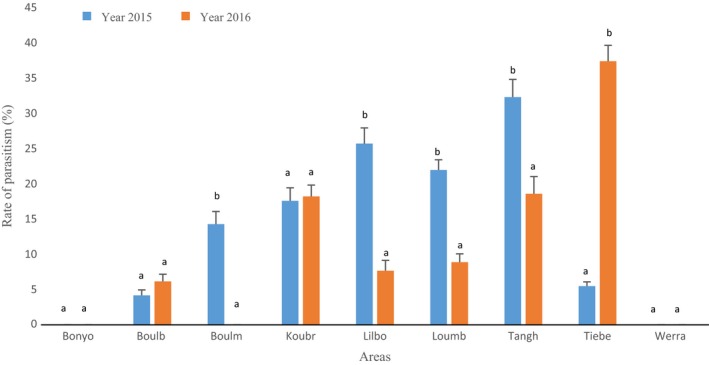
Average rate of parasitism (*Eretmocerus mundus and Encarsia vandrieschei*) in the different areas. Mean rates followed by the same letters are not significantly different (generalized Tukey's all‐pair comparisons test at *p* < .05). Areas: Boulm: Boulmiougou; Bonyo: Bonyolo; Boulb: Boulbi; Koubr: Koubri; Lilbo: Lilbouré; Loumb: Loumbila; Tangh: Tanghin; Tiebe: Tiebélé

Only two species of *B. tabaci* parasitoids were found, which are both hymenopteran wasps from the Aphelinidae family. One is *Eretmocerus mundus*, while the other, based on *mtCOI* sequences, is related to *Encarsia vandrieschei* (92% identity). *Encarsia* specimens were only found in one locality, Lilbouré. On the contrary, *E. mundus* was recorded in all the sites except in this locality. The rates of parasitism varied according to both the areas and years with a significant interaction (*F* = 48.53, *df* = 8, *p* value <2.10^−16^; Figure 6). In 2015, the rate of larval parasitism was higher in Tanghin and Lilbouré with a mean of 32.28% and 25.68% per plant, respectively, and the lowest was recorded in Boulbi and Tiébélé (4.17% and 5.49% per plant). In 2016, Koubri, Tanghin, and Tiébélé areas exhibited the highest rates of larval parasitism (18.19%, 18.58%, and 37.37%), and the lowest rate was recorded in Boulbi, Lilbouré, and Loumbila areas (6.15%, 7.67%, and 8.88% per plant). In Tiébélé, this rate increased greatly between 2015 and 2016: from 5.49% to 37.37% per plant.

**Figure 7:**
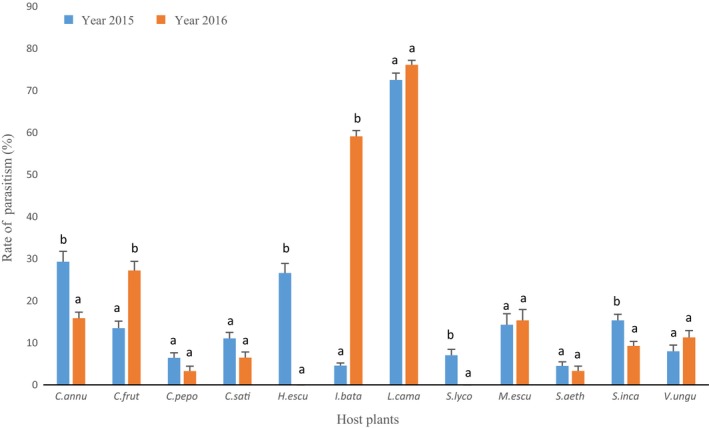
Average percentage of parasitism (*Encarsia vandrieschei* only on *Manihot esculenta* and *Eretmocerus mundus*) on the host plants. Mean rates followed by the same letters are not significantly different (generalized Tukey's all‐pair comparisons test at *p* < .05). Host plants: *C. annu: Capsicum annuum; C. frut: Capsicum frutescens, C. pepo: Cucurbita pepo; C. sati: Cucumis sativus; H. escu: Hibiscus esculentus; I. bata: Ipomoea batatas; L. cama: Lantana camara; S. lyco: Solanum lycopersicum; M. escu: Manihot esculenta; S. aeth: Solanum aethiopicum; S. inca: Solanum incanum; V. ungu: Vigna unguiculata*

There is also a significant interaction between the host plant and the year for the rate of parasitism (*F* = 35.7, *df* = 11, *p* value <2.10^−16^), even though, for the 2 years, the highest rate was observed on *L. camara* (72.52% in 2015, 76.13% in 2016). *I. batatas* presents one of the lowest rates in 2015, 4.60%, but a rate close to the rate observed on *L. camara* in 2016, 59.10% (Figure 7).

**Figure 8:**
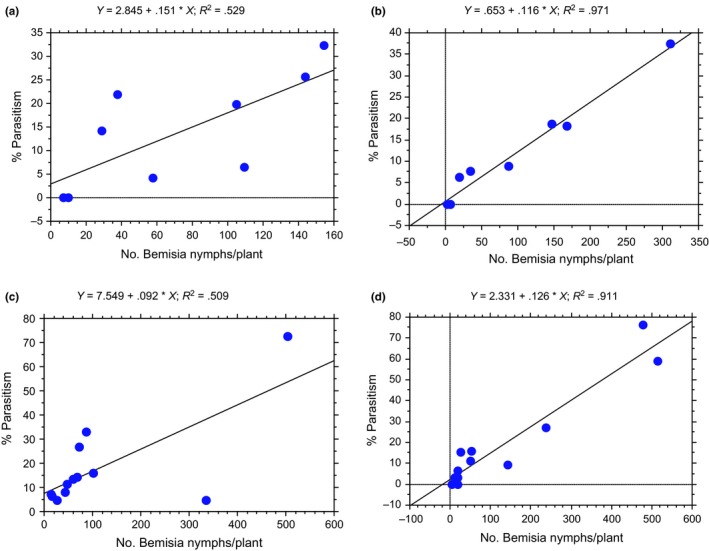
Relationship between *Bemisia tabaci* nymphs’ densities and rates of parasitism in the different areas in (a) 2015 and (b) 2016, and depending on the host plant in (c) 2015 and (d) 2016

Importantly, a clear relationship was observed between the rate of parasitism and the *B. tabaci* larval density. This was observed by locality (*p* = .02, *r* ^2^ = .53, in 2015 (Figure 8a); *p* < .0001, *r* ^2^ = .97, in 2016 (Figure 8b)) and by host plant (*p* = .009, *r* ^2^ = .51, in 2015 (Figure 8c); *p* < .0001, *r* ^2^ = .91, in 2016 (Figure 8d)).

## 4 DISCUSSION

The global analysis of *B. tabaci* and their parasitoids in nine localities of Burkina Faso gives valuable information on the level of biodiversity present in these areas including the abundance of whiteflies’ populations in connection with the host plant species, and the location, but also with the presence and abundance of parasitoids.

Before getting into the details, it is important to stress the high abundance of whiteflies (over 21 insects per plant) in seven areas sampled (Boulbi, Boulmiougou, Koubri, Lilbouré, Loumbila, Tanghin, and Tiébélé) in 2015. Moreover, this abundance increased in Koubri and Tiébélé from 2015 to 2016. These values are higher than those found by Zinga et al. (2013) in Central African Republic and Sseruwagi et al. (2004) in Uganda which, using the same sampling method, found on average 1.5 and 7.1 adults per plant, respectively. Our results provide compelling evidence that Burkina Faso is facing an upsurge of superabundant whiteflies. This might be linked to the continuous increase in the *B. tabaci* resistance to chemical products that has largely reduced the effectiveness of most available pesticides in Burkina Faso especially on cotton and vegetables plants (Gnankiné, Mouton, Savadogo et al., 2013).

Our results also give insights into the dynamics of biodiversity of this species complex. As previously reported (Gnankiné, Mouton, Henri et al., 2013; Gueguen et al., 2010), MED‐Q1, MED‐Q3, and ASL were detected in Burkina Faso. We also detected the sub‐Saharan African species SSA (biotype SSA2). SSA1 and SSA2 are the most widely distributed biotypes of SSA and occur in East, Central, South, and West Africa (Abdullahi, Atiri, Thottappilly, & Winter, 2004; Legg, French, Rogan, Okao‐Okuja, & Brown, 2002; Legg, Shirima et al., 2014; Tocko et al., 2017). SSA2 occurs in Cameroon, Benin, and Togo (Berry et al., 2004; Gnankiné, Mouton, Henri et al., 2013) and in the most southwestern part of the DRC (Legg, Sseruwagi et al., 2014). As previous surveys in Burkina Faso did not include cassava, it is probable that SSA2 was already present at that time. Globally, the diversity of *B. tabaci* in Burkina Faso is thus important and does not seem to have changed since our last survey in 2009.

MED‐Q1 was found to be highly dominant in most of the sampled sites and vegetable plants. It is sometimes found in sympatry (the same locality and the same plant) with ASL, suggesting the possible coexistence of the ASL and MED‐Q biotypes at least on a short timescale. Interestingly, in Lilbouré and Tiébélé where there were frequent insecticide applications, MED‐Q1 replaced SSA2 and ASL between 2015 and 2016. As MED‐Q1 is known for its ability to displace other biotypes, we can wonder whether this illustrates invasion/displacement in Burkina Faso. The high resistance of MED‐Q1 to various insecticides may favor its spreading in treated areas. In any case, this indicates that important modification of the local diversity of *B. tabaci* may occur from 1 year to another.

If invasion/displacements are frequent, then how is it possible to maintain local biodiversity in terms of species and/or biotypes? Host plant preference, host plant specialization, and plant‐dependent competitiveness could play a major role in the stability of *B. tabaci* communities. For example, SSA2 has only been found on cassava yet. An earlier survey in sub‐Saharan Africa (SSA1, SSA2, SSA3) (Abdullahi, Winter, Atiri, & Thottappilly, 2003) highlighted the fact that the populations that develop on cassava seem to be restricted to this host plant, whereas populations from other plants are polyphagous, but do not colonize cassava. Such niche specialization of the SSA, with cassava exercising a repulsive action on other biotypes, could allow its persistence even if other biotypes/species are present locally. Interestingly, we also collected MED‐Q1 adults, larvae, and nymphs on cassava. However, their success on this plant has not been determined yet, and it is also possible that SSA individuals can outcompete other *B. tabaci* species on this plant. This is probably what happens for MED‐Q3 on *L. camara*. Indeed, MED‐Q3 was the only biotype detected on this species and at extremely high density. Even the highly common MED‐Q1, which belongs to the same species, was not found on this plant. Interestingly, in Tunisia, MED‐Q1 has already been detected on *L. camara* (Saleh, Laarif, Clouet, & Gauthier, 2012), and MED‐Q1 individuals from Burkina Faso are able to develop on this species (Romba & Gnankiné, 2018). Altogether, this suggests that MED‐Q3 outcompetes other biotypes and species in this area on *L. camara*, which allow its local maintenance. On the opposite, very few MED‐Q3 individuals were detected on other plants than *L. camara*. This may be due to a strong specialization of this biotype to this plant, as suggested by its inability to develop on cotton in laboratory experiments (Romba & Gnankiné, 2018). Overall, only MED‐Q1 and ASL individuals seem to have similar realized niches.

Top‐down regulation could also play a role in maintaining biodiversity of *B. tabaci*. In the current investigations, two aphelinid parasitoid wasps were collected: *Eretmocerus mundus* and *Encarsia vandrieschei*. *Eretmocerus mundus* was recorded in all sites and host plants excepted in Lilbouré where *Encarsia* vandrieschei. was sampled, but only on cassava plants that hosted SSA individuals. This suggests that parasitoids could present some specificities to the *B. tabaci* species or the host plant. On the opposite*, Eretmocerus* was found to parasitize larvae of MED‐Q1, MED‐Q3, and ASL. A clear relationship between larval abundance and parasitism rate was recorded as those observed 12 years ago by Gnankiné (2005) and Otoidobiga, Vincent, and Stewart (2004). This confirms that parasitoids parasitize *B. tabaci* in a density‐dependent manner. This phenomenon could be explained by the production of allomones by plants. Indeed, it is known that the production of allomones is an indirect defense strategy of the plant that can attract parasitoids and predators (Mumm & Dicke, 2010). Most herbivore‐induced plant volatiles can be classified as terpenoids, green leaf volatiles, phenylpropanoids, and sulfur‐ or nitrogen‐containing compounds. Increased release of allomones by plants due to a higher *B. tabaci* abundance may thus attract more parasitoids and explain why the parasitism rate depends on whitefly abundance. It is however not clear how this phenomenon may interact with the local dynamics of *B. tabaci* community.

## 5 CONCLUSION

The present work has led to a better understanding of the distribution of whiteflies and their parasitoids in connection with host plants in Burkina Faso. Also, the *B. tabaci* biodiversity on vegetables, cassava, and ornamental plants in Burkina Faso has been updated. Among the four whitefly species/biotypes identified, one of them, MED‐Q1 biotype of *B. tabaci*, is currently a serious pest in Burkina Faso in view of its superabundance. Knowledge on the dynamics of *B. tabaci* biodiversity and its parasitoids each year is necessary for the development of effective control strategies as the different biological entities do not have the same insecticide resistance capacity. However, the important variations observed between the 2 years highlight that abundance of *B. tabaci* is highly fluctuant, which will probably impose regular adjustments in local control strategies.

## CONFLICT OF INTEREST

None declared.

## AUTHOR CONTRIBUTION

OG, RR, SFD, and FT contributed to the design of the study. VF, HH, and LM critically contributed to the implementation of the study. OG, RR, SFD, FT, HH, and LM conducted field evaluations and laboratory research. RR drafted the manuscript and OG, VF, HH, and LM revised it. All authors read and approved the final manuscript.
