# Supplementary material for: Abundance of Bemisia tabaci Gennadius (Hemiptera: Aleyrodidae) and its parasitoids on vegetables and cassava plants in Burkina Faso (West Africa)
Source: Ecol Evol. 2018 May 20;8(12):6091–103. doi: 10.1002/ece3.4078 (PMC6024141; doi:10.1002/ece3.4078)
Supplement: Supplementary file 1 [file ECE3-8-6091-s001.docx]

**B**

Fig. S1 (Supporting information): Relationship between whitefly abundance per area **(A)** and per plant **(B)** in 2015 and 2016.
