# Supplementary material for: Abundance of Bemisia tabaci Gennadius (Hemiptera: Aleyrodidae) and its parasitoids on vegetables and cassava plants in Burkina Faso (West Africa)
Source: Ecol Evol. 2018 May 20;8(12):6091–103. doi: 10.1002/ece3.4078 (PMC6024141; doi:10.1002/ece3.4078)
Supplement: Supplementary file 2 [file ECE3-8-6091-s002.docx]

Table SI (Supporting information): Frequencies of *Bemisia tabaci* biotypes related to host plants and localities.

| **Sample** | **Date collected** | **Locality** | **City** | **Host plant** | **Latin name** | **Family** | **^ƚ^Insecticides used (number of applications)** | **Biotypes or genetic groups present (%)^§^** |
| --- | --- | --- | --- | --- | --- | --- | --- | --- |
| TangO_E1 | 01/03/2016 | Tanghin | Ouagadougou | Eggplant | *Solanum incanum* | Solanaceae | OP(4), Pyr(4) | Q1(100) |
| TangO_E2 | 01/03/2016 |  |  | Cucumber | *Cucumis sativus* | Cucurbitaceae | Car(3) | Q1(80), ASL(20) |
| TangO_E3 | 01/03/2016 |  |  | Tomato | *Solanum lycopersicum* | Solanaceae | Pyr(4), Neo(4) | Q1(50), ASL(40), Q3(10) |
| TangO_E4 | 01/03/2016 |  |  | Local eggplant | *Solanum aethiopicum* | Solanaceae | Pyr(4), Aver(3) | Q1(100) |
| TangO_E5 | 01/03/2016 |  |  | Lantana camara | *Lantana camara* | Verbenaceae | - | Q3(100) |
| TangO_1 | 25/04/2015 |  |  | Potato | *Ipomoea batatas* | Convolvulaceae | OP(2) | ASL(100) |
| TangO_2 | 25/04/2015 |  |  | Eggplant | *Solanum incanum* | Solanaceae | OP(4) | Q1(100) |
| LoumO_E1 | 05/03/2016 | Loumbila | Ziniaré | Chilli pepper | *Capsicum frutescens* | Solanaceae | Neo(3), Pyr(3) | Q1(80), ASL(20) |
| LoumO_E2 | 05/03/2016 |  |  | Squash | *Cucurbita pepo* | Cucurbitaceae | Neo(2), Pyr(3) | Q1(100) |
| LoumO_E3 | 05/03/2016 |  |  | Okra | *Hibiscus esculentus* | Malvaceae | Pyr(2), OP(4) | Q1(100) |
| LoumO_E4 | 05/03/2016 |  |  | Local eggplant | *Solanum aethiopicum* | Solanaceae | Aver(3) | Q1(100) |
| LoumO_E5 | 05/03/2016 |  |  | Eggplant | *Solanum incanum* | Solanaceae | Pyr(4), Neo(4) | Q1(100) |
| LoumO_E6 | 05/03/2016 |  |  | Cucumber | *Cucumis sativus* | Cucurbitaceae | Neo(4), Pyr(4) | Q1(90), ASL(10) |

To be continued

Table SI continued.

| **Sample** | **Date collected** | **Locality** | **City** | **Host plant** | **Latin name** | **Family** | **^ƚ^Insecticides used (number of applications)** | **Biotypes or genetic groups present (%)^§^** |
| --- | --- | --- | --- | --- | --- | --- | --- | --- |
| LoumO_E7 | 05/03/2016 | Loumbila | Ziniaré | Tomato | *Solanum lycopersicum* | Solanaceae | Pyr(3), OP(1) | Q1(80), ASL(20) |
| LoumO_E8 | 08/05/2016 |  |  | Eggplant | *Solanum incanum* | Solanaceae | Pyr(4) | Q1(100) |
| LoumO_E9 | 08/05/2016 |  |  | Local eggplant | *Solanum aethiopicum* | Solanaceae | Pyr(4) | Q1(100) |
| BoulK_E1 | 06/03/2016 | Boulbi | Komsilga | Eggplant | *Solanum incanum* | Solanaceae | Neo(3), Pyr(3) | Q1(70), ASL(30) |
| BoulK_E2 | 06/03/2016 |  |  | Local eggplant | *Solanum aethiopicum* | Solanaceae | Pyr(4), OP(3) | Q1(80), ASL(20) |
| BoulK_E3 | 06/03/2016 |  |  | Squash | *Cucurbita pepo* | Cucurbitaceae | Pyr(4) | Q1(80), Q3(20) |
| BoulK_E4 | 06/03/2016 |  |  | Sweet pepper | *Capsicum annuum* | Solanaceae | Aver(2) | Q1(100) |
| BoulK_E5 | 06/03/2016 |  |  | Chilli pepper | *Capsicum frutescens* | Solanaceae | OP(3), Pyr(3) | Q1(100) |
| BoulK_E6 | 06/03/2016 |  |  | Cuncumber | *Cucumis sativus* | Cucurbitaceae | Aver(3) | Q1(90), Q3(10) |
| BoulK_1 | 13/06/2015 |  |  | Squash | *Cucurbita pepo* | Cucurbitaceae | Pyr(4), OP(4) | Q1(100) |
| BoulK_2 | 13/06/2015 |  |  | Squash | *Cucurbita pepo* | Cucurbitaceae | Pyr(4), OP(4) | Q1(100) |
| BoulO_E1 | 12/03/2016 | Boulmiougou | Ouagadougou | Tomato | *Solanum lycopersicum* | Solanaceae | Pyr(4), Neo(4) | Q1(90), ASL(10) |
| BoulO_E2 | 12/03/2016 |  |  | Eggplant | *Solanum incanum* | Solanaceae | Car(4) | Q1(100) |
| BoulO_E3 | 12/03/2016 |  |  | Sweet pepper | *Capsicum annuum* | Solanaceae | OP(4), Pyr(4) | Q1(100) |
| BoulO_E4 | 12/03/2016 |  |  | Cucumber | *Cucumis sativus* | Cucurbitaceae | Pyr(1) | Q1(80), Q3(20) |

To be continued

Table SI continued

| **Sample** | **Date collected** | **Locality** | **City** | **Host plant** | **Latin name** | **Family** | **^ƚ^Insecticides used(number of applications)** | **Biotypes or genetic groups present (%)^§^** |
| --- | --- | --- | --- | --- | --- | --- | --- | --- |
| BoulO_E5 | 12/03/2016 | Boulmiougou | Ouagadougou | Squash | *Cucurbita pepo* | Cucurbitaceae | Aver(1) | Q1(60), Q3(20), ASL(20) |
| BoulO_E6 | 12/03/2016 |  |  | Okra | *Hibiscus esculentus* | Malvaceae | Ben(1) | Q1(60), ASL(40) |
| BoulO_1 | 19/05/2015 |  |  | Okra | *Hibiscus esculentus* | Malvaceae | Ben(1) | Q1(80), ASL(20) |
| TiebP_E1 | 19/03/2016 | Tiebélé | Pô | Chilli pepper | *Capsicum frutescens* | Solanaceae | Pyr (4) | Q1(100) |
| TiebP_E3 | 19/03/2016 |  |  | Potato | *Ipomea batatas* | Convolvulaceae | Aver(4), SI (3) | Q1(100) |
| TiebP_E4 | 19/03/2016 |  |  | Cowpea | *Vigna unguiculata* | Fabaceae | Neo(4) | Q1(100) |
| TiebP_E5 | 19/03/2016 |  |  | Sorrel | *Hibiscus sabdarifa* | Malvaceae | Pyr (4) | Q1(100) |
| TiebP_E6 | 19/03/2016 |  |  | Eggplant | *Solanum incanum* | Solanaceae | Pyr (4) | Q1(100) |
| TiebP_E7 | 19/03/2016 |  |  | Cassava | *Manihot esculenta* | Euphorbiaceae | - | Q1(100) |
| TiebP_E8 | 19/03/2016 |  |  | Okra | *Hibiscus esculentus* | Malvaceae | Neo (4) | Q1(100) |
| TiebP_E9 | 19/03/2016 |  |  | Jatropha | *Jatropha gossypiifolia* | Euphorbiaceae | - | Q1(100) |
| TiebP_E10 | 19/03/2016 |  |  |  | *Physalis angulata* | Solanaceae | - | Q1(100) |
| TiebP_1 | 25/05/2015 |  |  | Potato | *Ipomoea batatas* | Convolvulaceae | OP(4), Pyr(4), Neo (4) | ASL(100) |
| TiebP_2 | 25/05/2015 |  |  | Green bean | *Phaseolus vulgaris* | Fabaceae | OP(4), Pyr(4), Neo (4) | ASL(100) |
| LiboB_E1 | 21/03/2016 | Lilbouré | Bazega | Eggplant | *Solanum incanum* | Solanaceae | Pyr(4) | Q1(100) |
| LiboB_E3 | 21/03/2016 |  |  | Cassava | *Manihot esculenta* | Euphorbiaceae | - | AnSL2(100) |
| LiboB_E4 | 21/03/2016 |  |  | Squash | *Cucurbita pepo* | Cucurbitaceae | Neo(4), Pyr(4) | Q1(100) |
| LiboB_E5 | 21/03/2016 |  |  | okra | *Hibiscus esculentus* | Malvaceae | APG(2) | Q1(100) |

To be continued

Table SI continued.

| **Sample** | **Date collected** | **Locality** | **City** | **Host plant** | **Latin name** | **Family** | **^ƚ^Insecticides used(number of applications)** | **Biotypes or genetic groups present (%)^§^** |
| --- | --- | --- | --- | --- | --- | --- | --- | --- |
| LiboB_E6 | 21/03/2016 | Lilbouré | Bazega | Potato | *Ipomoea batatas* | Convolvulaceae | Pyr(4) | Q1(100) |
| LiboB_1 | 12/06/2015 |  |  | cassava | *Manihot esculenta* | Euphorbiaceae | - | Q1(10), AnSL2(90) |
| KoubK_E1 | 23/03/2016 | Koubri | Kombissiri | Sweet pepper | *Capsicum annuum* | Solanaceae | Neo(4), Pyr(4) | Q1(100) |
| KoubK_E2 | 23/03/2016 |  |  | Tomato | *Solanum lycopersicum* | Solanaceae | Pyr(2) | Q1(90), ASL(10) |
| KoubK_E3 | 23/03/2016 |  |  | Eggplant | *Solanum incanum* | Solanaceae | Neo(4), Pyr(2) | Q1(100) |
| KoubK_E4 | 23/03/2016 |  |  | Chilli pepper | *Capsicum frutescens* | Solanaceae | Pyr(2) | Q1(100) |
| KoubK_E5 | 23/03/2016 |  |  | Squash | *Cucurbita pepo* | Cucurbitaceae | Pyr(2) | Q1(100) |
| KoubK_1 | 11/06/2015 |  |  | Chilli pepper | *Capsicum frutescens* | Solanaceae | Neo(4), Pyr(4) | Q1(100) |
| KoubK_2 | 11/06/2015 |  |  | Local eggplant | *Solanum aethiopicum* | Solanaceae | Pyr(4) | Q1(90), Q3(10) |
| KoubK_3 | 11/06/2015 |  |  | Eggplant | *Solanum incanum* | Solanaceae | Pyr(4) | Q1(100) |
| KoubK_4 | 11/06/2015 |  |  | Squash | *Cucurbita pepo* | Cucurbitaceae | SI(1) | Q1(90), Q3(10) |
| Werr_E1 | 25/03/2016 | Werra | Koudougou | Tomato | *Solanum lycopersicum* | Solanaceae | OP(1), Pyr(1) | Q1(40), ASL(60) |
| Werr_E2 | 25/03/2016 |  |  | Eggplant | *Solanum incanum* | Solanaceae | Pyr(2), Neo(1) | Q1(90), ASL(10) |
| Werr_E3 | 25/03/2016 |  |  | Local eggplant | *Solanum aethiopicum* | Solanaceae | OP(1), Pyr(2), Aver(1) | Q1(80), ASL(20) |
| BonyR_E1 | 26/03/2016 | Bonyolo | Réo | Local eggplant | *Solanum aethiopicum* | Solanaceae | Pyr(1), OP(1) | Q1(100) |
| BonyR_E2 | 26/03/2016 |  |  | Chilli pepper | *Capsicum frutescens* | Solanaceae | Aver(2) | Q1(100) |
| BonyR_E3 | 26/03/2016 |  |  | Eggplant | *Solanum incanum* | Solanaceae | Pyr(2) | Q1(100) |

^ƚ^This column indicates whether pesticides are used or not, Pyrethrinoid (Pyr) ; Organophosphorus (OP) ; Neonicotinoid (Neo) ; Avermectins (Aver) ; Benzoylurea (Ben) ; Aminophosphonates glycine(APG) ; Inorganic substance (SI). **^§^**Percentage of each biotype found at the collecting site.
